# A Chewing Gum Containing *Heyndrickxia coagulans* SNZ1969^®^ and Volatile Sulphur Compounds: An Exploratory Secondary Analysis of a Double-Blind Randomized Controlled Trial

**DOI:** 10.3390/nu18162735

**Published:** 2026-08-21

**Authors:** Silvia Cirio, Giacomo Mantegazza, Claudia Salerno, Simone Guglielmetti, Aesha Allam, Guglielmo Campus, Maria Grazia Cagetti

**Affiliations:** 1 Department of Biomedical, Surgical and Dental Sciences, University of Milan, Via Beldiletto 1, 20142 Milan, Italy; claudia.salerno@ymail.com (C.S.); aesha.allam@unimi.it (A.A.); maria.cagetti@unimi.it (M.G.C.); 2 Department of Biotechnology and Biosciences, University of Milano-Bicocca, Piazza Della Scienza 2, 20126 Milan, Italy; giacomo.mantegazza@hest.ethz.ch (G.M.); simone.guglielmetti@unimib.it (S.G.); 3 Department of Cariology, Institute of Odontology, Sahlgrenska Academin, University of Gothenburg, 413 90 Gothenburg, Sweden; guglielmo.giuseppe.campus@gu.se; 4 Department of Oral and Maxillofacial Sciences, Sapienza University of Rome, 00161 Rome, Italy; 5 Department of Cariology, Saveetha, Dental College and Hospitals, SIMATS, Chennai 600077, India

**Keywords:** *Heyndrickxia coagulans*, probiotic, halitosis, bad breath, oral microbiome, chewing gum, metataxonomics, volatile sulphur compounds, hydrogen sulphide

## Abstract

**Background**: Chewing gum may reduce oral malodor through salivary stimulation and may also serve as a carrier for oral probiotics. Evidence on *Heyndrickxia coagulans* in this setting is lacking. **Methods**: This exploratory secondary analysis used data from a randomized, blinded, placebo-controlled study in which 52 healthy adults were assigned to a sugar-free gum with *H. coagulans* SNZ1969^®^ or to a matched placebo gum for four weeks. Hydrogen sulphide (H_2_S) and methyl mercaptan (CH_3_SH) were measured by OralChroma™ at baseline (T_0_), during intervention (T_1_–T_2_), and one week after gum discontinuation (T_3_). Dental plaque 16S rRNA profiles from the parent trial were re-analysed in relation to VSC values. **Results**: Forty-four participants completed follow-up. H_2_S and CH_3_SH concentrations did not differ between probiotic and placebo groups at any visit, and changes from baseline were also similar between arms. In the probiotic group, H_2_S was lower than baseline at T_2_ (*p* = 0.008) and T_3_ (*p* = 0.031), but this within-arm decrease was not greater than the change seen with placebo. Taxon-level analyses suggested nominal associations with taxa plausibly linked to sulphur metabolism, mainly for H_2_S, but no association survived false-discovery-rate correction. **Conclusions**: The probiotic gum did not show a statistically significant additional effect on VSC concentrations compared with the placebo gum. The within-arm H_2_S decrease and microbiome signals are exploratory and require confirmation in trials designed for participants with clinically established halitosis.

## 1. Introduction

Halitosis, commonly referred to as bad breath, is a widespread oral condition affecting nearly 30% of the global adolescent and adult population. Its prevalence seems to be greater in low- and middle-income countries, and it may lead to considerable social discomfort and psychological distress [1,2,3,4]. It is described as an unpleasant odor arising from the oral cavity, with causes that may be intra-oral or extra-oral/systemic. In approximately 80–90% of cases, halitosis originates intra-orally and is primarily associated with microbial degradation of organic substrates such as desquamated epithelial cells, saliva proteins, and food debris [1,5]. The metabolic activity of these microorganisms leads to the generation of volatile sulfur compounds (VSCs), particularly hydrogen sulfide (H_2_S) and methyl mercaptan (CH_3_SH), which are widely regarded as the principal causes of oral malodor [2]. Elevated levels of CH_3_SH have been strongly associated with periodontal inflammation; by contrast, H_2_S is primarily related to tongue coating and the metabolic activity of anaerobic bacteria colonizing the dorsal surface of the tongue [3]. Since clinical studies have demonstrated that tongue coating, periodontal pockets and dental plaque are significantly correlated with VSCs levels and that mechanical tongue cleaning can reduce oral malodor [3,6], contemporary management strategies for halitosis focus on reducing the bacterial load, limiting substrate availability, neutralizing VSCs, and modulating the oral microbiota [1].

Chewing gum has been widely investigated as an adjunctive approach for controlling oral malodor. Clinical studies have shown that chewing gum can transiently reduce oral malodor and VSCs concentrations, largely due to increased salivary secretion and mechanical cleansing effects [7,8,9]. To enhance efficacy, various functional compounds have been incorporated into chewing gum formulations, such as plant extracts, e.g., green tea [10] or eucalyptus extract [11] or sodium bicarbonate [12,13]. In addition, probiotic-based strategies have emerged as a promising approach for the management of halitosis.

Probiotics refer to live microorganisms capable of promoting host health when administered at appropriate doses [14]. Evidence from clinical studies suggests that specific probiotic strains, such as *Streptococcus salivarius* K12, *Weissella cibaria* CMU, and *Ligilactobacillus salivarius* WB21, may reduce VSCs production and improve halitosis-related parameters [15,16,17,18,19,20,21,22,23].

Chewing gum may represent an interesting vehicle for probiotic delivery, as it can allow extended contact between the microorganism and oral tissues while also stimulating salivary flow [24]. Previous studies evaluating probiotic-containing chewing gums have reported reductions in salivary mutans streptococci and improvements in selected oral-health-related parameters [25,26]. Furthermore, clinical evidence suggests that probiotic chewing gum may contribute to reductions in oral malodor [7,27].

Formerly classified as *Bacillus coagulans*, *Heyndrickxia coagulans* is an endospore-forming, lactic acid-producing bacterium whose combination of industrial robustness and well-established safety has made it a valuable microorganism for various probiotic applications [28]. A growing body of research has evaluated the contribution of *H. coagulans* to oral health, with studies focusing on conditions such as dental caries, gingivitis, and periodontitis [29]. To date no study has specifically investigated the potential impact of *H. coagulans* delivered through chewing gum on VSCs levels as markers of oral malodor. This gap in the literature is noteworthy, especially considering the increasing interest in probiotic-based products for managing oral malodor. The present manuscript reports a secondary exploratory analysis of a randomized clinical trial whose primary microbiome outcomes have been previously published. While the original publication focused on dental biofilm ecology and microbiome composition, the current analysis specifically investigates volatile sulphur compounds (H_2_S and CH_3_SH) and their associations with microbial taxa, with the aim of exploring potential microbiological mechanisms involved in oral malodor. The novelty of the present analysis lies not in re-describing global microbiome changes, which have been reported elsewhere, but in exploring whether VSC dynamics are associated with specific dental plaque taxa potentially involved in sulphur metabolism. Therefore, the aim of this study was to investigate whether a probiotic chewing gum containing *H. coagulans* could modulate oral VSCs concentrations, particularly H_2_S and CH_3_SH, in a randomized controlled trial. In addition, using dental plaque 16S rRNA gene profiling data generated in the same trial and previously reported for biofilm ecology outcomes, we performed an exploratory secondary analysis focused on VSCs-associated oral microbial taxa.

## 2. Materials and Methods

### 2.1. Design of the Study

The present manuscript reports a secondary analysis of the same randomized, double-blind, placebo-controlled trial previously described by Cirio et al. [30], focusing specifically on oral VSCs and their exploratory associations with dental plaque microbial taxa. It was conducted at the Department of Biomedical, Surgical and Dental Sciences, University of Milan (Milan, Italy), in accordance with the principles of the Declaration of Helsinki (University of Milan Ethics Committee; protocol no. 24/24; 13 February 2024, Supplementary File S1). This study was registered post hoc at ISRCTN Registry (n. ISRCTN13055033; 12 April 2025). The CONSORT 2010 checklist for reporting randomized trials is provided in the Supplementary Material (Supplementary File S2).

### 2.2. Sample Selection

The study enrolled healthy adult volunteers selected from the staff of Perfetti Van Melle SpA (PVM, Lainate, Italy) and from students attending the degree program in Dental Hygiene and in Dentistry and in the postgraduate program in Pediatric Dentistry of the University of Milan (Milan, Italy).

Because no prior data was available to estimate the expected effect of *H. coagulans* chewing gum on oral VSCs concentrations, no formal sample-size calculation was performed for the present secondary endpoint. The present analyses should therefore be considered exploratory and hypothesis-generating. The original trial enrolled 52 participants to ensure an adequate sample for exploratory clinical and microbiome analyses while accounting for potential dropouts.

Details regarding the inclusion and exclusion criteria, chewing gum production, and the chewing gum administration protocol have been previously described by Cirio et al. [30] and are reported in the Supplementary Materials (Supplemetary Files S3 and S4).

A total of 56 individuals responded to the recruitment advertisement. Of these, two declined to participate because they anticipated difficulties complying with the study protocol. After providing written informed consent, the remaining volunteers underwent a screening interview to assess eligibility according to the predefined inclusion and exclusion criteria, followed by a clinical examination performed by a calibrated dentist (SC). Clinical assessments included the Plaque Index [31], Gingival Index [32], and stimulated salivary flow rate. Two additional participants were excluded because of a suspected allergy to components of the chewing gum. Consequently, 52 eligible participants were enrolled and randomly assigned, using a computer-generated randomization sequence, to either the probiotic group (*n* = 26) or the placebo group (*n* = 26). Allocation was concealed, and both participants and investigators remained blinded throughout the study.

### 2.3. Outcomes Assessment

**Figure 1:**
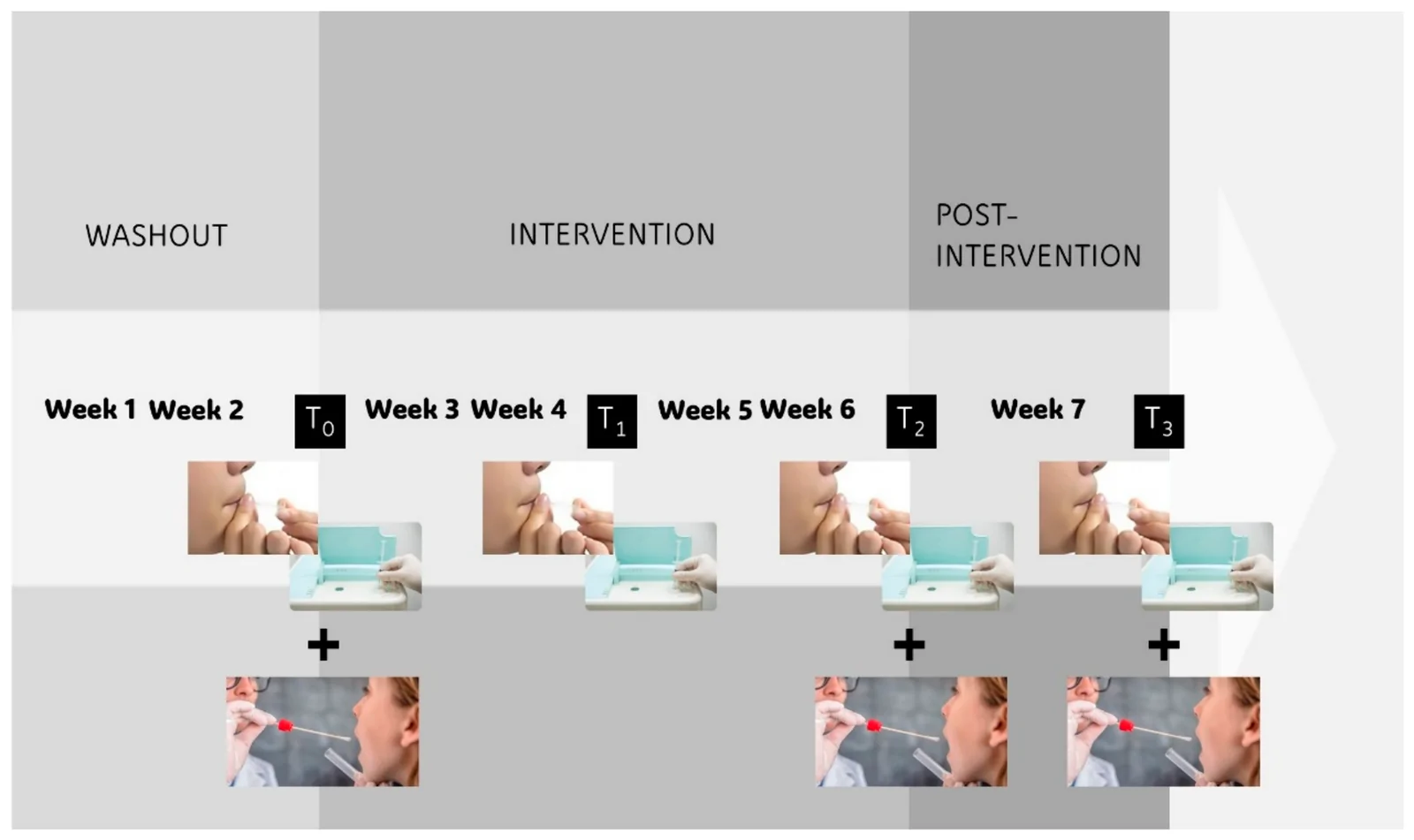
Sample collection timeline.

VSC measurements were performed at T_0_ (following the 2-week washout period and prior to the start of the intervention), at T_1_ and T_2_ (two and four weeks after intervention onset, respectively), and at T_3_ (one week after completion of the intervention). Plaque samples were collected at T_0_, T_2_ and T_3_ follow up (Figure 1).

VSCs in oral air samples were measured using a halitometer (OralChroma^™^, INSISTEC, Barcelona, Spain). Oral breath samples were collected using sterile single-use needleless syringes with a capacity of 1 mL. Participants were instructed to take a deep breath, place the tip of the sterile syringe inside the mouth, seal their lips around it, and keep it in position for 30 s. Care was taken to avoid contamination of the syringe with saliva. After the waiting period, the operator manipulated the syringe plunger to collect 1 mL of oral air, which was then immediately injected into the device for analysis. All assessments were performed between 8:00 and 10:00 a.m.

Before testing, participants were instructed to avoid garlic, onion, and other strong-flavored condiments, and to limit the consumption of margarine, milk, fried foods, sardines, salami, mortadella, sausages, red meat, cheese, sulphur-containing foods (e.g., cabbage, broccoli, cauliflower, and eggs), and alcohol. Participants were also asked not to brush their teeth during the 24 h preceding the test and to refrain from consuming candies or chewing gum on the morning of the assessment. H_2_S and CH_3_SH concentrations were selected for analysis because of their stronger association with oral health, in accordance with previous studies [33,34]. VSC-based halitosis was recorded when H_2_S levels were ≥112 ppb and/or CH_3_SH levels were ≥26 ppb.

Dental plaque 16S rRNA gene profiling data used in the present manuscript were generated within the same randomized controlled trial previously reported by Cirio et al. [30]. In the primary publication, these data were analyzed to assess the impact of *H. coagulans* SNZ1969^®^ chewing gum on dental biofilm ecology, including global microbiome composition, diversity metrics, and taxonomic profiling. The present study re-used these data for an exploratory secondary analysis focused on associations between dental plaque microbial taxa and VSCs concentrations. Dental plaque sampling and microbiome analysis were described previously by Cirio et al. [30] and reported in the Supplementary File S5.

Since the generation and primary ecological analysis of these sequencing data have already been reported, for the present manuscript H_2_S and CH_3_SH concentrations were considered clinical exploratory outcomes. Microbiome–VSCs associations were considered secondary exploratory outcomes.

### 2.4. Statistical Analysis

Descriptive statistics were calculated for all variables and reported as mean ± standard deviation (SD) or median and interquartile range, as appropriate. The Shapiro–Wilk test was used to assess the normality of continuous data. Homoscedasticity was assessed when required for parametric comparisons. For between-group comparisons of continuous variables, the Mann–Whitney U test was applied due to non-normal data distributions. For categorical variables, Fisher’s exact test was used to evaluate differences between groups. Intra-group comparisons of repeated measures (T_0_, T_1_, T_2_, T_3_) were performed using the Wilcoxon signed-rank test for paired data. In specific subgroup analyses, Welch’s *t*-test was applied when assumptions for equal variances were violated. A *p*-value < 0.05 was considered statistically significant. All statistical analyses were conducted using Stata/MP 19.0 for Windows.

Baseline associations between microbial taxa and VSCs (H_2_S and CH_3_SH) were evaluated using Spearman correlation on centered log-ratio (CLR)-transformed abundances across multiple taxonomic levels. To account for multiple testing, *p*-values were adjusted using the Benjamini–Hochberg false discovery rate (FDR) procedure within each endpoint and taxonomic level. Both nominal *p*-values and FDR-adjusted *p*-values were considered, with FDR-adjusted *p*-values < 0.05 interpreted as statistically significant. The effect of probiotic supplementation on VSCs-associated taxa over time was assessed using longitudinal regression models with subject-clustered robust standard errors, adjusting for visit, treatment group, age, and sex. Treatment-by-visit interactions were further examined within a difference-in-differences framework. In addition, between-group differences in changes in CLR-transformed abundances (ΔT_2_–T_0_ and ΔT_3_–T_0_) were analyzed using Mann–Whitney U tests.

Given the exploratory nature of the microbiome-VSCs analyses and the limited sample size, results from taxon-level analyses were interpreted as hypothesis-generating, particularly when statistical significance did not persist after FDR correction.

## 3. Results

### 3.1. Sample Characteristics

The study was conducted from September 2024 to April 2025. A total of 52 volunteers, equally divided between the probiotic and placebo groups (26 participants per group), completed the 2-week washout period. At the end of this phase, three participants assigned to the probiotic group were excluded: one due to poor adherence to the study protocol and two because of intercurrent health conditions. Therefore, the chewing gum intervention was initiated in 23 participants in the probiotic group and 26 participants in the placebo group.

**Figure 2:**
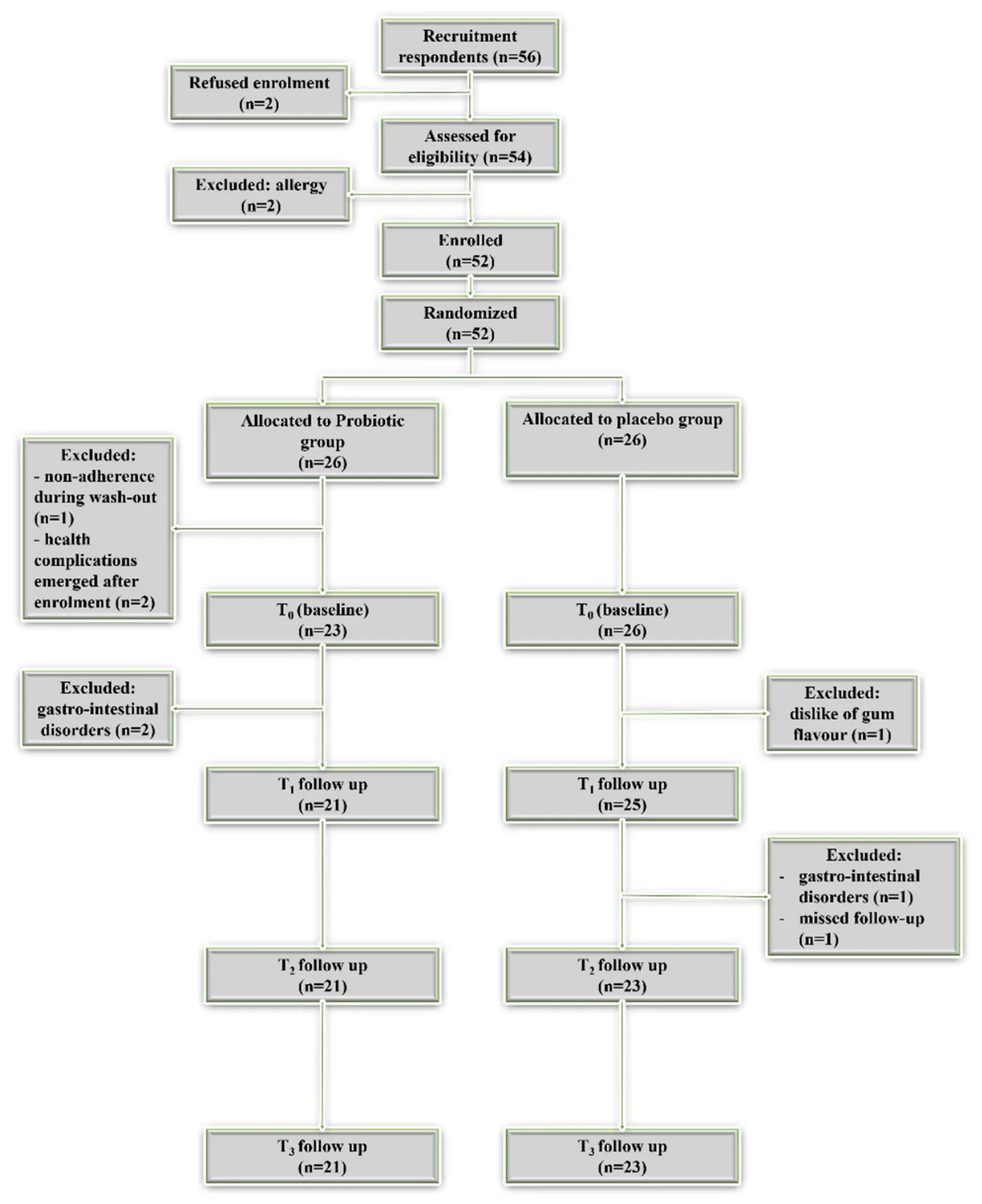
Flow diagram of participant recruitment, randomization, and follow-up in the clinical trial.

During the intervention period, one participant in the placebo group withdrew before the first follow-up assessment (T_1_) due to taste intolerance, while two participants in the probiotic group discontinued the study because of gastrointestinal adverse effects. Before the second follow-up assessment (T_2_), two additional participants from the placebo group were excluded, one due to a gastrointestinal disorder and the other because of a missed study visit. Overall, 21 participants in the probiotic group and 23 participants in the placebo group completed the study, resulting in an overall dropout rate of 13.5%. A full flow diagram is reported in Figure 2.

Characteristics of participants, compliance and adverse effects reported in the two study groups during the experimental period have been reported in a previous study [30].

Final analyses included 44 participants, 21 in probiotic arm and 23 in placebo arm (mean age 28.4 years; 86.4% female), with no significant differences in age or sex between groups.

### 3.2. VSCs Concentrations

At baseline, no significant differences in H_2_S or CH_3_SH levels were observed between groups (Table 1).

At T_0_, six participants in both the probiotic and placebo groups exhibited H_2_S levels ≥112 ppb, whereas eight participants in both groups exhibited CH_3_SH levels ≥26 ppb.

**Figure 3:**
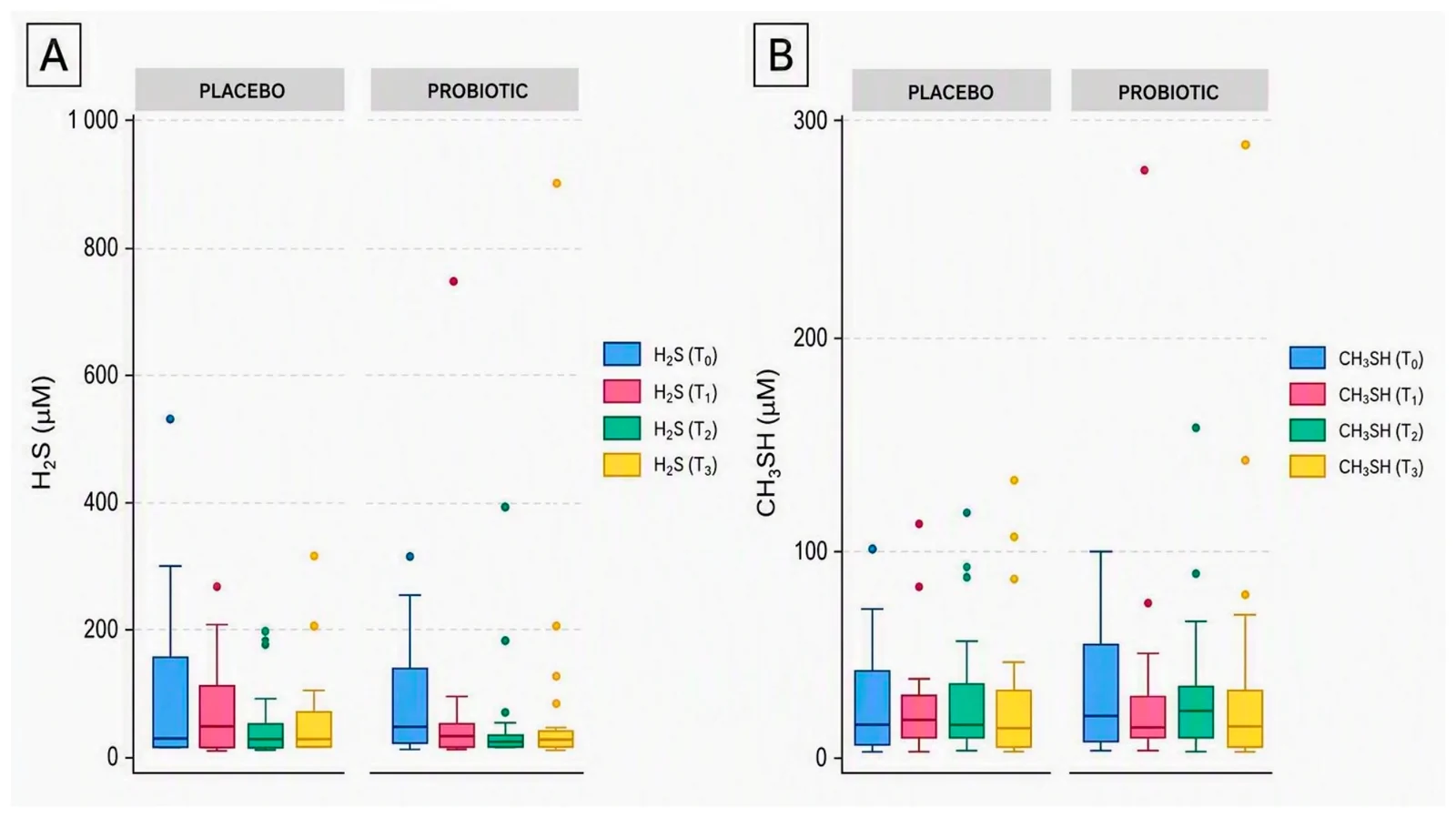
Box plots of H_2_S (**A**) and CH_3_SH (**B**) concentrations (ppb) at baseline (T_0_) and follow-up time points (T_1_–T_3_) in the placebo and probiotic groups.

Both H_2_S and CH_3_SH showed a general tendency to decrease over time, although the pattern was clearer for H_2_S than for CH_3_SH. H_2_S values decreased within the probiotic group, with significantly lower values at T_2_ and T_3_ compared with baseline (*p* = 0.008 and *p* = 0.031, respectively). Median values at these time points approached zero (Table 1, Figure 3A). However, between-group comparisons did not show statistically significant differences at individual time points. For the primary between-group contrast, the observed difference in mean ΔH_2_S from T_0_ to T_2_ between the probiotic and placebo groups was −3.90 ppb, with an approximate 95% CI of −62.32 to 54.51 ppb.

CH_3_SH showed a similar but less marked pattern, with no significant intra-group differences at any time point in either group (Table 1, Figure 3B). No statistically significant between-group differences were observed for longitudinal changes in either H_2_S or CH_3_SH over time (Table 1), nor were any significant differences found in the reductions in values between follow-up intervals when comparing the probiotic and placebo groups (Table 2).

### 3.3. Previously Reported Global Microbiome Analyses

Global microbiome analyses from this randomized trial, previously reported elsewhere, did not reveal substantial differences in alpha diversity, beta diversity or overall taxonomic composition between probiotic and placebo groups following treatment. These findings are summarized in Supplementary Files S6 and S7 and are consistent with the absence of statistically significant between-group differences observed for VSC concentrations in the present secondary analysis.

### 3.4. Microbial Associations with VSCs (Baseline)

No taxa remained significantly associated with baseline VSCs levels after correction for multiple testing using the Benjamini–Hochberg false discovery rate (FDR) procedure. All FDR-adjusted *p*-values were > 0.05. Therefore, all microbiome findings reported below should be considered exploratory and hypothesis-generating and should not be interpreted as confirmatory evidence of associations between microbial taxa and VSC concentrations (Table 3).

For H_2_S, nominal positive associations were observed with *Kingella* and an uncultured *Prevotellaceae* genus, while nominal negative associations were found for *Atopobium* spp., including *A. rimae*, and *Veillonellaceae*. For CH_3_SH, *Porphyromonas gulae* was nominally positively associated, whereas *Johnsonella ignava* and *Veillonellaceae* showed nominal negative associations. None of these associations remained significant after FDR correction. These taxa were considered for exploratory interpretation based on biological plausibility in the oral ecosystem.

### 3.5. Longitudinal Microbial Modulation

Exploratory longitudinal models identified multiple taxa nominally associated with VSCs and potentially modulated by treatment. A higher number of signals was observed for H_2_S-associated taxa compared with CH_3_SH-associated taxa (Supplementary File S8).

Taxa showing both VSCs association and nominal treatment-related changes with coherent directionality were further examined. Overall, signals were more consistent for H_2_S-related taxa.

**Figure 4:**
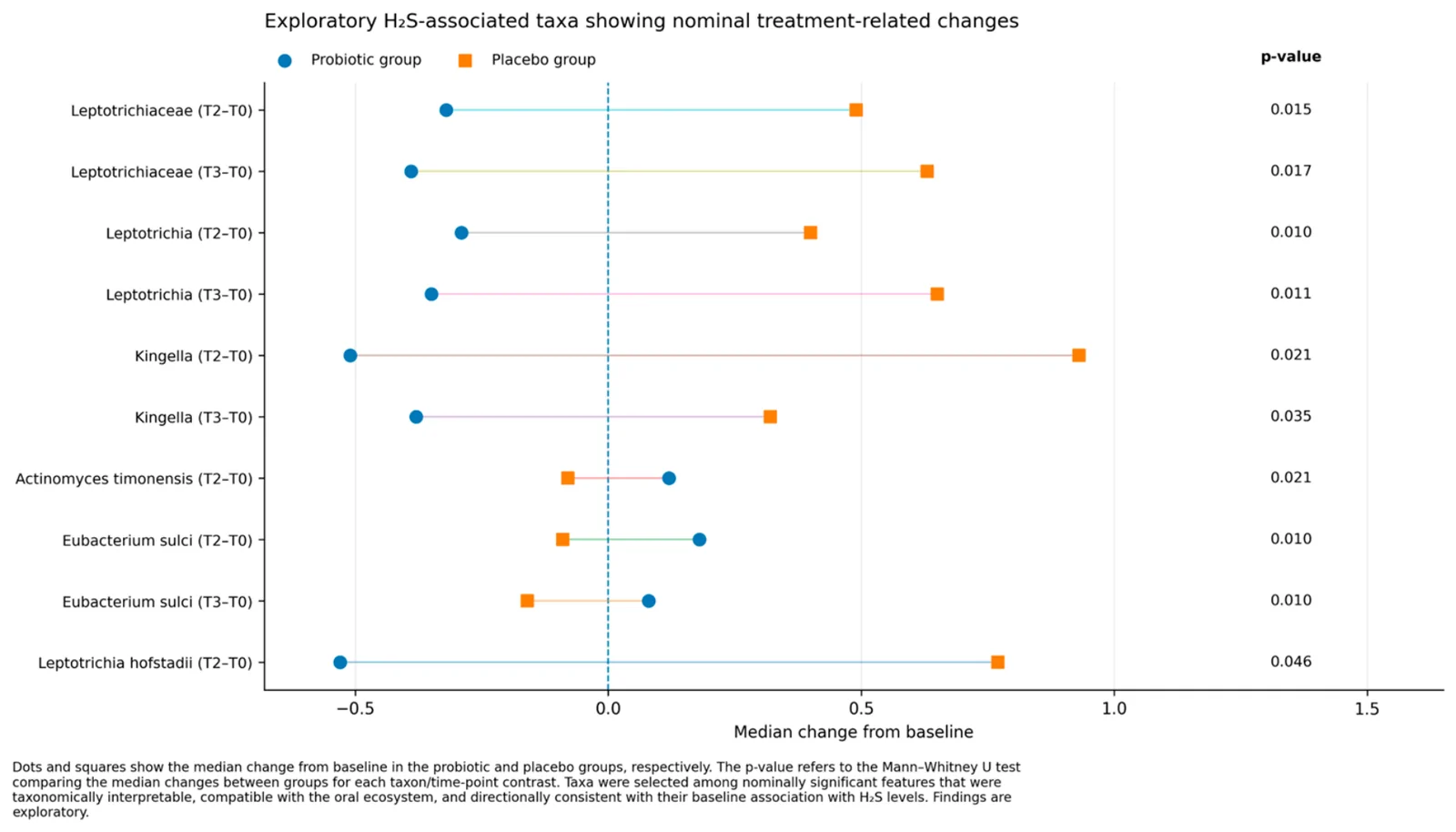
Exploratory H_2_S-associated taxa with nominal treatment-related changes.

Among the most biologically plausible taxa, members of *Leptotrichiaceae* (*Leptotrichia* spp. and *L. hofstadii*) and *Kingella* showed reductions in the probiotic group and increases in controls. Conversely, *Actinomyces timonensis* and *Eubacterium sulci* showed the opposite pattern (Figure 4; Supplementary File S9).

These directional changes were consistent with their baseline associations with H_2_S, suggesting a possible probiotic-related modulation of taxa nominally associated with H_2_S. However, these findings should be interpreted exclusively as exploratory and hypothesis-generating, as no taxon-level association remained statistically significant after correction for multiple testing. The taxa reported in Figure 4 and Supplementary File S9 were selected based on nominal significance, taxonomic interpretability, biological plausibility, and coherent directionality with baseline VSCs associations.

## 4. Discussion

This randomized, double-blind, placebo-controlled trial evaluated the effects of a chewing gum containing *Heyndrickxia coagulans* SNZ1969^®^ on oral VSCs and oral microbiota composition. Unlike the primary publication arising from this clinical trial, which focused on global changes in dental biofilm ecology, the present secondary analysis specifically explored VSC dynamics and their relationships with microbial taxa potentially involved in sulphur metabolism. Overall, no statistically significant between-group differences were observed for either H_2_S or CH_3_SH concentrations. Nevertheless, the probiotic group showed significant within-group reductions in H_2_S and several exploratory microbiome signals, which may provide hypotheses for future adequately powered studies.

The previously reported alpha-diversity and beta-diversity analyses further support this interpretation. No major differences in global microbial diversity or overall community structure were observed between treatment groups throughout the study period. This lack of microbiome-wide separation is consistent with the absence of statistically significant between-group differences in VSCs concentrations. Taken together, these findings suggest that any potential probiotic-related effects were likely subtle and confined to specific ecological components rather than reflecting a broad reorganization of the dental plaque microbiota. The absence of a clear separation in community structure suggests that any probiotic-related effects were not associated with large-scale microbiome reorganization but rather with subtle ecological shifts involving specific taxa.

From a conceptual perspective, this comparison assesses whether the probiotic strain provides an additional effect beyond that of the chewing gum vehicle alone, in which chewing gum acts as both delivery system and modulator of salivary flow and biofilm dynamics. Within this framework, the oral microbiome may be considered as a dynamic ecological network in which transient perturbations may alter functional outputs such as VSCs production rather than inducing stable compositional shifts.

Both study arms involved chewing gum use; therefore, the observed reductions in VSCs likely reflect, at least in part, the established effects of mastication on salivary stimulation and mechanical clearance [7,35]. However, the present design does not allow isolation of the specific contribution of chewing per se versus probiotic supplementation. Accordingly, all between-group differences should be interpreted as the additional effect of *H. coagulans* over a shared chewing gum vehicle, rather than as a direct comparison between masticatory and non-masticatory conditions.

Within this context, both groups exhibited reductions in VSCs over time, consistent with the established effects of chewing gum on salivary stimulation and oral clearance. Nevertheless, the probiotic group showed statistically significant within-group reductions in H_2_S concentrations at both T_2_ and T_3_, whereas no comparable statistically significant reductions were observed in the placebo group. Although these changes were not significantly different from those observed between groups, they may represent an exploratory signal worthy of further investigation. Notably, H_2_S values increased between T_2_ and T_3_ following discontinuation of the intervention, indicating a partial rebound toward baseline levels. This observation suggests that any potential effect may be transient and not fully maintained after cessation of chewing gum administration. H_2_S is considered one of the principal contributors to intra-oral malodor and is often associated with microbial activity occurring on the tongue dorsum and within supragingival biofilms. Therefore, even modest reductions in H_2_S could be biologically relevant, particularly in populations presenting elevated baseline VSC levels. However, because the present study enrolled predominantly orally healthy individuals who were not selected for clinically confirmed halitosis or elevated baseline VSC levels and was not powered to detect between-group differences, the clinical significance of these observations remains uncertain and should be interpreted cautiously. An alternative explanation is that part of the observed reduction may reflect non-specific effects of chewing gum use, such as increased salivary flow and mechanical clearance of volatile sulphur compounds. Because H_2_S values partially rebounded after treatment discontinuation, the relative contribution of probiotic-related ecological effects and chewing-related effects cannot be disentangled in the present study and deserves further investigation in future trials.

These findings are consistent with previous probiotic studies in halitosis, which more frequently report reductions in H_2_S than CH_3_SH, including interventions using *Lactobacillus*, *Streptococcus*, and *Weissella species* [36].

The present study adds preliminary evidence by evaluating *H. coagulans* delivered via chewing gum, a delivery system that ensures repeated oral exposure while simultaneously stimulating salivary flow The delayed pattern of effect observed over time is compatible with the hypothesis that repeated exposure to the probiotic may influence oral ecological dynamics and VSC-related microbial activity [37].

To facilitate biological interpretation, a subset of nominally significant taxa was examined according to predefined ecological plausibility criteria. Because none of these findings remained significant after correction for multiple-testing, the observations discussed below should be interpreted exclusively as exploratory and hypothesis-generating. The observed pattern of nominal microbiome changes was directionally coherent with the reduction in H_2_S observed within the probiotic group. In particular, taxa positively associated with H_2_S, such as Leptotrichia, Leptotrichiaceae and Kingella, tended to decrease in participants receiving the probiotic, whereas taxa negatively associated with H_2_S, including Actinomyces timonensis and [Eubacterium] sulci, tended to increase. These findings should not be considered confirmatory evidence, but they provide a biologically plausible ecological framework that may help explain the observed reduction in H_2_S concentrations and support future hypothesis-driven investigations [38,39].

From a clinical perspective, the potential relevance of the observed H_2_S reduction should be interpreted in light of the characteristics of the study population. Most participants were orally healthy adults and were not selected on the basis of clinically diagnosed halitosis or elevated baseline VSC levels. Consequently, the magnitude of any achievable reduction was inherently limited by a floor effect. In subjects with established halitosis and higher baseline H_2_S concentrations, a comparable ecological modulation might potentially translate into a larger clinical benefit. However, this hypothesis requires confirmation in specifically designed clinical trials.

*H. coagulans* may have exerted transient ecological pressure through competitive interactions and modulation of local environmental conditions, potentially influencing microbial metabolic activity related to VSC production. The chewing gum matrix likely enhances these effects by prolonging oral exposure and increasing salivary flow, thereby affecting substrate availability and biofilm turnover [35]. However, these mechanisms remain hypothetical and were not directly assessed in this study.

Strengths of the study include its randomized double-blind design, repeated longitudinal sampling, targeted quantification of VSCs, and integration of clinical and microbiome data. Baseline comparability and high completion rates further support internal validity. Importantly, the combined interpretation of VSCs dynamics and microbiome shifts provides a preliminary multi-level framework for generating hypotheses on the ecological modulation of oral malodor markers.

Several limitations should be acknowledged. First, all microbiome analyses were exploratory and involved multiple comparisons without formal adjustment, and therefore should be interpreted as hypothesis-generating rather than confirmatory. In addition, the trial was originally designed to investigate dental biofilm ecology rather than as a therapeutic intervention for halitosis, and the present manuscript reports a secondary exploratory analysis of that dataset. Consequently, participants were not recruited on the basis of clinically confirmed halitosis or elevated VSC concentrations. Baseline VSC concentrations were generally low, which may have limited the magnitude of measurable reductions, particularly for CH_3_SH. This potential floor effect should be considered when interpreting the absence of significant between-group differences. Moreover, the study was not specifically powered to detect between-group differences in volatile sulphur compound concentrations. For the primary between-group comparison, the observed difference in mean ΔH_2_S from T_0_ to T_2_ between the probiotic and placebo groups was −3.90 ppb, with an approximate 95% CI of −62.32 to 54.51 ppb. With the available sample size, the study had 80% power to detect a standardized between-group effect of approximately Cohen’s d = 0.86, corresponding to a minimum detectable difference of approximately 84 ppb for ΔH_2_S from T_0_ to T_2_. Because these analyses were conducted as a secondary exploratory investigation and VSC outcomes were not included in the original sample-size calculation, the relatively small sample size and substantial inter-individual variability may have reduced the ability to identify smaller but potentially relevant treatment-related differences. Consequently, the absence of statistically significant between-group differences should not be interpreted as evidence of absence of effect. Furthermore, because the study population consisted predominantly of orally healthy adults, the findings should not be directly extrapolated to patients with clinically confirmed halitosis.

Another limitation is that trial registration was completed after participant recruitment and follow-up had been initiated. Although VSC assessments were prospectively planned and included in the Ethics Committee-approved protocol, retrospective registration may increase the perceived risk of selective reporting and should therefore be acknowledged when interpreting the findings.

Future studies specifically enrolling subjects with elevated baseline VSC levels and incorporating multi-omics approaches, such as metatranscriptomics or metabolomics, may better clarify the functional mechanisms underlying probiotic-mediated modulation of oral malodor.

## 5. Conclusions

Within the limitations of this exploratory secondary analysis, no statistically significant between-group differences were observed in VSC concentrations following administration of *H. coagulans* SNZ1969^®^ chewing gum. Although significant within-group reductions in H_2_S were observed in the probiotic arm, these changes were not significantly different from those observed in the placebo group. Likewise, several microbiome signals were identified, but none remained significant after correction for multiple testing. Therefore, the findings should be considered hypothesis-generating rather than confirmatory and should not be interpreted as evidence of probiotic efficacy. Larger adequately powered trials are required to determine whether *H. coagulans* provides benefits beyond those attributable to the chewing gum vehicle itself.

Furthermore, because participants were not selected on the basis of clinically confirmed halitosis, the present findings should not be generalized to patients with established oral malodor without further confirmatory studies.

## Figures and Tables

**Table 1:** VSCs (H_2_S and CH_3_SH) values at baseline (T_0_), after 14 and 28 days from the beginning of administration (T_1_ and T_2_), and 7 days after the end of administration (T_3_) in the probiotic and placebo groups. Values are reported as mean (SD); median (IQR).

Variable	Probiotic (*n* = 21)	Placebo (*n* = 23)	*p*-Value
	Mean (SD); Median (IQR)		
**H_2_S (ppb)**			
T_0_	76.81 (94.34); 30 (6–126)	73.39 (124.54); 12 (0–93.5)	0.394 ^b^
T_1_	53.67 (154.82); 16 (1–36)	66.74 (79.45); 34 (6–84.5)	0.070 ^b^
T_2_	36.90 (87.16); 6 (0–20)	37.39 (57.51); 13 (2–33)	0.327 ^b^
T_3_	67.62 (189.15); 12 (2–23)	43.17 (71.12); 12 (0–55.5)	0.868 ^b^
T_0_ vs. T_1_	0.120 ^c^	0.637 ^c^	
T_0_ vs. T_2_	0.008 ^c^	0.088 ^c^	
T_0_ vs. T_3_	0.031 ^c^	0.437 ^c^	
**CH_3_SH (ppb)**			
T_0_	28.05 (30.62); 16 (4–52)	22.65 (26.15); 12 (3–35)	0.787 ^b^
T_1_	29.57 (56.98); 11 (6–27)	20.52 (25.55); 14 (6.5–22.5)	1.000 ^b^
T_2_	27.29 (35.07); 19 (6–31)	25.26 (31.03); 12 (6.5–32)	0.787 ^b^
T_3_	35.10 (65.40); 12 (2–29)	23.78 (34.64); 11 (1.5–28)	0.689 ^b^
T_0_ vs. T_1_	0.728 ^c^	0.749 ^c^	
T_0_ vs. T_2_	0.835 ^c^	0.704 ^c^	
T_0_ vs. T_3_	0.917 ^c^	1.000 ^c^	

N: number; SD: standard deviation; IQR: interquartile range. Normality and heteroskedasticity of continuous data were assessed with Shapiro–Wilk test. Comparisons between groups were performed using Mann–Whitney U test (^b^); Wilcoxon signed-rank test (^c^).

**Table 2:** Changes (Δ) in VSCs levels from baseline to different time points across groups. Values are reported as mean (SD); median (IQR).

	Probiotic (*n* = 21)	Placebo (*n* = 23)	*p* Value
	Mean (SD); Median (IQR)		
**H_2_S (ppb)**			
Δ T_1_–T_0_	−23.14 (130.87); −11 (−63–10)	−6.65 (99.06); 2 (−17–32.5)	0.165
Δ T_2_–T_0_	−39.90 (74.87); −13 (−46–0)	−36.00 (114.10); −6 (−67.5–1)	0.541
Δ T_3_–T_0_	−9.19 (144.27); −20 (−56–3)	−30.22 (142.18); −3 (−21.5–10)	0.398
**CH_3_SH (ppb)**			
Δ T_1_–T_0_	1.52 (50.02); 0 (−13–11)	−2.13 (32.57); 2 (−9–10)	0.912
Δ T_2_–T_0_	−0.76 (34.28); 0 (−11–12)	2.61 (40.80); 2 (−17.5–16)	0.604
Δ T_3_–T_0_	7.05 (47.89); −3 (−8–12)	1.13 (32.78); −1 (−8.5–12)	0.968

N: number; SD: standard deviation; IQR: interquartile range. Normality and heteroskedasticity of continuous data were assessed with Shapiro–Wilk test. Between-group differences were evaluated using the Mann–Whitney U test.

**Table 3:** Association between taxa and VSCs levels assessed using Spearman correlation. Nominal *p*-values are reported. No association remained statistically significant after FDR correction; therefore, the taxa listed should be interpreted as nominal, exploratory findings.

Full_Taxonomy	Rho	Nominal *p*-Value
**CH_3_SH**		
*p_Actinobacteriota;c_Actinobacteria;o_Bifidobacteriales;f_Bifidobacteriaceae*	−0.34	0.025
*p_Firmicutes;c_Negativicutes;o_Veillonellales-Selenomonadales;f_Veillonellaceae;g_Veillonellaceae*	−0.33	0.028
*p_Firmicutes;c_Clostridia;o_Lachnospirales;f_Lachnospiraceae;g_Johnsonella;s_Johnsonella_ignava*	−0.41	0.006
*p_Bacteroidota;c_Bacteroidia;o_Bacteroidales;f_Porphyromonadaceae;g_Porphyromonas;s_gulae*	0.37	0.013
*p_Patescibacteria;c_Gracilibacteria;o_JGI_0000069-P22;f_JGI_0000069-P22;g_JGI_0000069-P22;s_*Candidatus*_Gracilibacteria*	0.36	0.017
*p_Actinobacteriota;c_Actinobacteria;o_Propionibacteriales;f_Propionibacteriaceae;g_Pseudopropionibacterium;s_propionicum*	0.34	0.024
*p_Firmicutes;c_Negativicutes;o_Veillonellales-Selenomonadales;f_Veillonellaceae;g_Veillonellaceae;s_*unidentified	−0.34	0.025
**H_2_S**		
*p_Firmicutes;c_Negativicutes;o_Veillonellales-Selenomonadales;f_Veillonellaceae;g_Veillonellaceae*	−0.37	0.014
*p_Firmicutes;c_Clostridia;o_Peptostreptococcales-Tissierellales;f_Anaerovoracaceae;g_Amnipila*	−0.33	0.027
*p_Firmicutes;c_Clostridia;o_Lachnospirales;f_Lachnospiraceae;g_Butyrivibrio*	−0.32	0.037
*p_Proteobacteria;c_Gammaproteobacteria;o_Burkholderiales;f_Neisseriaceae;g_Kingella*	0.31	0.041
*p_Actinobacteriota;c_Coriobacteriia;o_Coriobacteriales;f_Atopobiaceae;g_Atopobium*	−0.31	0.041
*p_Bacteroidota;c_Bacteroidia;o_Bacteroidales;f_Prevotellaceae;g_uncultured*	0.30	0.046
*p_Firmicutes;c_Negativicutes;o_Veillonellales-Selenomonadales;f_Veillonellaceae;g_Veillonellaceae;s_*unidentified	−0.38	0.012
*p_Actinobacteriota;c_Coriobacteriia;o_Coriobacteriales;f_Atopobiaceae;g_Atopobium;s_Atopobium_rimae*	−0.33	0.027
*p_Firmicutes;c_Clostridia;o_Peptostreptococcales-Tissierellales;f_Anaerovoracaceae;g_Amnipila;s_*uncultured_organism	−0.33	0.027
*p_Firmicutes;c_Clostridia;o_Peptostreptococcales-Tissierellales;f_Anaerovoracaceae;g_[Eubacterium]_nodatum_*group*;s_sulci*	−0.30	0.048

## Data Availability

The data supporting the findings of this study are publicly available in the University of Milan Dataverse repository at the following DOI: https://doi.org/10.13130/RD_UNIMI/VATL7W. All datasets generated and analyzed during the current study have been deposited and can be accessed.

## Supplementary Materials

The following supporting information can be downloaded at: https://www.mdpi.com/article/10.3390/nu18162735/s1, S1. English translation of the relevant extract from the Ethics Committee-approved protocol; S2. CONSORT 2010 checklist of information to include when reporting a randomized trial; S3. Inclusion and exclusion criteria; S4. Production and use of chewing gum; S5. Dental plaque sampling and microbiome analysis; S6. Alpha-diversity analyses (previously reported in the primary publication arising from the same randomized clinical trial); S7. Beta-diversity analysis (PCoA) (previously reported in the primary publication arising from the same randomized clinical trial); S8. Nominally VSCs-associated taxa; S9. Exploratory H_2_S-associated taxa showing nominal treatment-related changes with coherent directionality. Taxa were selected among nominally significant features that were taxonomically interpretable, compatible with the oral ecosystem, and directionally consistent with their baseline association with H_2_S levels.

## Abbreviations

The following abbreviations are used in this manuscript:

VSCs	Volatile sulphur compounds
H_2_S	Hydrogen sulphide
CH_3_SH	Methyl mercaptan
ppb	Parts per billion
CFU	Colony-Forming Units
ppm	Parts Per Million
DNA	Deoxyribonucleic Acid
rRNA	Ribosomal Ribonucleic Acid
FDR	False Discovery Rate
CLR	Centered log-ratio
SD	Standard deviation

## References

[1] Porter S.R., Scully C. (2006). Oral Malodour (Halitosis). BMJ.

[2] Tonzetich J. (1977). Production and Origin of Oral Malodor: A Review of Mechanisms and Methods of Analysis. J. Periodontol..

[3] Yaegaki K., Sanada K. (1992). Volatile Sulfur Compounds in Mouth Air from Clinically Healthy Subjects and Patients with Periodontal Disease. J. Periodontal Res..

[4] Quirynen M., Dadamio J., Van Den Velde S., De Smit M., Dekeyser C., Van Tornout M., Vandekerckhove B. (2009). Characteristics of 2000 Patients Who Visited a Halitosis Clinic. J. Clin. Periodontol..

[5] Villa A., Zollanvari A., Alterovitz G., Cagetti M., Strohmenger L., Abati S. (2014). Prevalence of Halitosis in Children Considering Oral Hygiene, Gender and Age. Int. J. Dent. Hyg..

[6] Seemann R., Kison A., Bizhang M., Zimmer S. (2001). Effectiveness of Mechanical Tongue Cleaning on Oral Levels of Volatile Sulfur Compounds. J. Am. Dent. Assoc..

[7] Muniz F.W.M.G., Friedrich S.A., Silveira C.F., Rösing C.K. (2017). The Impact of Chewing Gum on Halitosis Parameters: A Systematic Review. J. Breath Res..

[8] Rösing C.K., Gomes S.C., Bassani D.G., Oppermann R.V. (2009). Effect of Chewing Gums on the Production of Volatile Sulfur Compounds (VSC) In Vivo. Acta Odontol. Latinoam..

[9] Hattab F.N. (2023). Chewing Gum for Oral and Dental Health: A Review. J. Oral Health Community Dent..

[10] Zeng Q.C., Wu A.Z., Pika J. (2010). The Effect of Green Tea Extract on the Removal of Sulfur-Containing Oral Malodor Volatiles *in Vitro* and Its Potential Application in Chewing Gum. J. Breath Res..

[11] Tanaka M., Toe M., Nagata H., Ojima M., Kuboniwa M., Shimizu K., Osawa K., Shizukuishi S. (2010). Effect of Eucalyptus-Extract Chewing Gum on Oral Malodor: A Double-Masked, Randomized Trial. J. Periodontol..

[12] Wild J.E., Nelson B.J., Hubbard M.E., Bowman J.P. (2001). Oral Malodor Control Afforded through the Use of Sodium Bicarbonate-Containing Chewing Gum. Compend. Contin. Educ. Dent..

[13] Wåler S.M. (1997). The Effect of Zinc-Containing Chewing Gum on Volatile Sulfur-Containing Compounds in the Oral Cavity. Acta Odontol. Scand..

[14] Hill C., Guarner F., Reid G., Gibson G.R., Merenstein D.J., Pot B., Morelli L., Canani R.B., Flint H.J., Salminen S. (2014). The International Scientific Association for Probiotics and Prebiotics Consensus Statement on the Scope and Appropriate Use of the Term Probiotic. Nat. Rev. Gastroenterol. Hepatol..

[15] Bolos A., Bolos O.C., Maghet E., Danila A.I., Briceag R., Bumbu B.A. (2026). Clinical and Microbiological Effects of *Streptococcus salivarius* K12 Lozenges and Zinc Mouthrinse on Persistent Intra-Oral Halitosis. Microorganisms.

[16] He L., Yang H., Chen Z., Ouyang X. (2020). The Effect of *Streptococcus salivarius* K12 on Halitosis: A Double-Blind, Randomized, Placebo-Controlled Trial. Probiotics Antimicrob. Proteins.

[17] Burton J.P., Chilcott C.N., Moore C.J., Speiser G., Tagg J.R. (2006). A Preliminary Study of the Effect of Probiotic *Streptococcus salivarius* K12 on Oral Malodour Parameters. J. Appl. Microbiol..

[18] Lee S.-K., Lim K.-O., Yang K.-I., You S.-J., Kang M.-S., Lee W.-P. (2026). Oral Probiotic *Weissella cibaria* Improves Halitosis and Reduces Periodontal Pathogens: A Randomized, Double-Blind, Placebo-Controlled Trial. BMC Oral Health.

[19] Han H., Yum H., Cho Y.-D., Kim S. (2023). Improvement of Halitosis by Probiotic Bacterium *Weissella cibaria* CMU: A Randomized Controlled Trial. Front. Microbiol..

[20] Passadakis G., Neophytou C., Davidopoulou S., Papadimitriou K. (2025). Effectiveness of Probiotics in Managing Oral Halitosis: A Systematic Review of Randomized Controlled Trials. J. Int. Soc. Prev. Community Dent..

[21] Lee D.-S., Lee S.-A., Kim M., Nam S.-H., Kang M.-S. (2020). Reduction of Halitosis by a Tablet Containing *Weissella cibaria* CMU: A Randomized, Double-Blind, Placebo-Controlled Study. J. Med. Food.

[22] Iwamoto T., Suzuki N., Tanabe K., Takeshita T., Hirofuji T. (2010). Effects of Probiotic *Lactobacillus salivarius* WB21 on Halitosis and Oral Health: An Open-Label Pilot Trial. Oral Surg. Oral Med. Oral Pathol. Oral Radiol. Endodontology.

[23] Motta P.D.B., Gonçalves M.L.L., Gallo J.M.A.S., Sobral A.P.T., Motta L.J., Mayer M.P.A., Kawamoto D., De Andrade D.C., Santos E.M., Fernandes K.P.S. (2024). Short Term Effect of Antimicrobial Photodynamic Therapy and Probiotic *L. salivarius* WB21 on Halitosis: A Controlled and Randomized Clinical Trial. PLoS ONE.

[24] Cirio S., Salerno C., Guglielmetti S.D., Mezzasalma V., Sarrica A., Kirika N., Campus G., Cagetti M.G. (2025). In Vivo Study on the Salivary Kinetics of Two Probiotic Strains Delivered via Chewing Gum. Microorganisms.

[25] Caglar E., Kavaloglu S.C., Kuscu O.O., Sandalli N., Holgerson P.L., Twetman S. (2007). Effect of Chewing Gums Containing Xylitol or Probiotic Bacteria on Salivary Mutans Streptococci and Lactobacilli. Clin. Oral Investig..

[26] Ericson D., Hamberg K., Bratthall G., Sinkiewicz-Enggren G., Ljunggren L. (2013). Salivary IgA Response to Probiotic Bacteria and Mutans Streptococci after the Use of Chewing Gum Containing *Lactobacillus reuteri*. Pathog. Dis..

[27] Keller M.K., Bardow A., Jensdottir T., Lykkeaa J., Twetman S. (2012). Effect of Chewing Gums Containing the Probiotic Bacterium *Lactobacillus reuteri* on Oral Malodour. Acta Odontol. Scand..

[28] Jurenka J. (2012). *Bacillus coagulans*: Monograph. Altern. Med. Rev. J. Clin. Ther..

[29] Cirio S., Campus G., Salerno C., Allam A., Cagetti M.G. (2026). Oral Health Benefits of *Heyndrickxia coagulans*: A Systematic Review and Meta-Analysis of Current Evidence. Front. Oral Health.

[30] Cirio S., Mantegazza G., Salerno C., Guglielmetti S., Allam A., Campus G., Cagetti M.G. (2026). Assessing the Impact of *Heyndrickxia coagulans* Administered Through Sugar-Free Chewing Gum on Dental Biofilm: A Double-Blind Randomized Controlled Trial. Nutrients.

[31] Silness J., Loe H. (1964). Periodontal disease in pregnancy. II. Correlation between oral hygiene and periodontal condtion. Acta Odontol. Scand..

[32] Löe H., Silness J. (1963). Periodontal Disease in Pregnancy I. Prevalence and Severity. Acta Odontol. Scand..

[33] Tangerman A. (2002). Halitosis in Medicine: A Review. Int. Dent. J..

[34] Tangerman A., Winkel E.G. (2008). The Portable Gas Chromatograph OralChroma^TM^: A Method of Choice to Detect Oral and Extra-Oral Halitosis. J. Breath Res..

[35] Wessel S.W., Van Der Mei H.C., Maitra A., Dodds M.W.J., Busscher H.J. (2016). Potential Benefits of Chewing Gum for the Delivery of Oral Therapeutics and Its Possible Role in Oral Healthcare. Expert Opin. Drug Deliv..

[36] Tangerman A., Winkel E.G. (2007). Intra- and Extra-Oral Halitosis: Finding of a New Form of Extra-Oral Blood-Borne Halitosis Caused by Dimethyl Sulphide. J. Clin. Periodontol..

[37] Perotti S., Mantegazza G., Pierallini E., Kirika N., Duncan R., Telesca N., Sarrica A., Guglielmetti S. (2024). Human in Vivo Assessment of the Survival and Germination of *Heyndrickxia coagulans* SNZ1969 Spores Delivered via Gummy Candies. Curr. Res. Food Sci..

[38] Takahashi N. (2015). Oral Microbiome Metabolism: From “Who Are They?” to “What Are They Doing?”. J. Dent. Res..

[39] Lamont R.J., Koo H., Hajishengallis G. (2018). The Oral Microbiota: Dynamic Communities and Host Interactions. Nat. Rev. Microbiol..

