# Supplementary material for: A Chewing Gum Containing Heyndrickxia coagulans SNZ1969® and Volatile Sulphur Compounds: An Exploratory Secondary Analysis of a Double-Blind Randomized Controlled Trial
Source: Nutrients. 2026 Aug 21;18(16):2735. doi: 10.3390/nu18162735 (PMC13516665; doi:10.3390/nu18162735)
Supplement: Supplementary file 1 [file nutrients-18-02735-s001.zip › nutrients-4436851-supplementary.pdf]

A Chewing Gum Containing Heyndrickxia coagulans SNZ1969® and Volatile Sulphur Compounds: An Exploratory Secondary Analysis of a Double-Blind Randomized Controlled Trial

Supplementary file

S1. English translation of the relevant extract from the Ethics Committee-approved protocol

S2. CONSORT 2010 checklist of information to include when reporting a randomized trial.

S3. Inclusion and exclusion criteria.

S4. Production and use of chewing gum.

S5. Dental plaque sampling and microbiome analysis.

S6. Alpha-diversity analyses (previously reported in the primary publication arising from the same randomized clinical trial).

S7. Beta-diversity analysis (PCoA) (previously reported in the primary publication arising from the same randomized clinical trial).

S8. Nominally VSCs-associated taxa.

S9. Exploratory H_2S-associated taxa showing nominal treatment-related changes with coherent directionality. Taxa were selected among nominally significant features that were taxonomically interpretable, compatible with the oral ecosystem, and directionally consistent with their baseline association with H2S levels.

S1. English translation of the relevant extract from the Ethics Committee-approved protocol

This supplementary file provides an English translation of the relevant sections of the Ethics Committee-approved study protocol, dated 13 February 2024. The translated extract includes the study aim, eligibility criteria, intervention, outcome measures, timing of assessments, dental plaque and tongue coating sampling procedures, microbiome analysis, and VSC assessment.

Research proposal

"In vivo evaluation of the salivary release of three different probiotic strains administered through chewing gum and their ability to colonize the oral cavity."

Aim of the study

The aim of this study is to determine the ability of sugar-free chewing gums containing, alternatively, *Lactobacillus rhamnosus*, *Weizmannia coagulans* or *Lactobacillus reuteri* to release probiotics into the oral cavity following chewing and, if release occurs, to investigate their ability to colonize the oral cavity.

Study design

The study is conceived as an experimental food-intervention study with a microbiological endpoint and consists of two phases:

- Phase 1: pilot study evaluating the release of probiotics into saliva after administration through chewing gum;
- Phase 2: evaluation of the colonization of hard and soft oral surfaces by probiotics administered through chewing gum.

Study population

Potential participants will be recruited among employees of Perfetti Van Melle S.p.A. and students of the Degree Course in Dentistry and Dental Prosthetics, the Degree Course in Dental Hygiene, and the Postgraduate School in Paediatric Dentistry at the University of Milan.

Inclusion criteria

Participants will be eligible if they meet the following criteria:

1. age between 18 and 65 years;
2. at least 24 teeth, excluding third molars;
3. healthy subjects with Gingival Index ≤ 2 and Plaque Index ≤ 2;
4. stimulated salivary flow between 1.5 and 2.0 mL/min.

Exclusion criteria

Exclusion criteria will be:

1. presence of systemic diseases;
2. pregnancy or breastfeeding;
3. history of drug abuse;

4. smoking;
5. fixed orthodontic appliances.

Intervention

All chewing gums tested will be produced and supplied by Perfetti Van Melle S.p.A. The chewing gums will be sugar-free.

The chewing gums will alternatively contain one of the following probiotic strains, at a concentration of 0.2 × 10^9 CFU:

1. *Lactobacillus rhamnosus* GG in free form;
2. *Lactobacillus rhamnosus* GG in microencapsulated form;
3. *Weizmannia coagulans* SNZ1969;
4. *Lactobacillus reuteri.*

Phase 2

The chewing gum showing the best performance in Phase 1 will be used as the treatment in Phase 2.

The study will have a randomized controlled two-arm design:

- subjects in the study group will use chewing gum containing the probiotic strain;
- subjects in the placebo group will use chewing gum identical in ingredients, taste, colour and size to that used in the study group, but without the probiotic strain.

All enrolled subjects will receive home oral hygiene instructions and will be provided with a toothbrush and fluoride toothpaste, 1450 ppm F. Participants will be asked not to use any mouthwash or other oral hygiene product during the experimental period.

The study will last seven weeks and will include:

- a two-week washout phase;
- a four-week chewing gum administration phase;
- a one-week follow-up phase.

During the four-week administration phase, participants will be asked to consume the assigned product five times per day — in the morning after breakfast, mid-morning, after lunch, mid-afternoon and in the evening — at least 30 minutes after toothbrushing, for 30 consecutive days.

Outcomes

The primary outcome will be the colonization of dental plaque and tongue surface by probiotic microorganisms.

Secondary outcomes will be:

- the degree of biodiversity of the microbial community of dental plaque and tongue coating;
- the assessment of volatile sulphur compounds, VSCs, emitted in exhaled breath.

Timing of sample collection and VSC assessment

Dental plaque samples, tongue coating samples and VSC analysis will be collected/performed at the following time points:

- T0: after the two-week washout period, before starting the administration period;
- T1: after two weeks of administration;
- T2: after four weeks of administration;
- T3: one week after the end of the administration period.

Dental plaque and tongue coating sampling

Tongue coating samples will be collected using a sterile swab rubbed over the dorsal surface of the tongue. The samples will be placed in sterile tubes containing Tris-EDTA.

Dental plaque samples will be collected from the buccal surfaces of teeth 1.6, 1.1, 2.6 and 3.1 and from the lingual surfaces of teeth 3.6 and 4.6. Sampling sites will be isolated with sterile cotton rolls and collection will be performed using sterile periodontal curettes. The samples will be placed in tubes containing Tris-EDTA.

The tubes will be labelled, stored at a temperature between 0 and 4 °C, and transported to the laboratory within four hours of collection. Upon receipt, samples will be immediately transferred to −80 °C until batch analysis.

Microbiological and metataxonomic analyses

Total DNA will be extracted from each sample using the PowerLyzer PowerSoil DNA Isolation Kit. DNA will be used for:

1. qPCR analysis, using a pair of primers specific for the probiotic strain under study, to detect and quantify its presence in the samples;
2. metataxonomic analysis, based on 16S rRNA gene profiling, consisting of sequencing of the V3–V4 region of the 16S rRNA gene. Sequencing reads will be analysed using the QIIME 2 bioinformatics pipeline, with the Greengenes database for taxonomic assignment.

VSC assessment

Volatile sulphur compounds emitted in exhaled breath will be measured using a halimeter, OralChroma™. All subjects will be tested between 8:00 and 11:00 a.m. They will be asked to avoid garlic, onion and other seasonings, and to limit the consumption of margarine, milk, fried potatoes, sardines, salami, mortadella, sausage, red meat, cheese, sulphur-containing foods such as cabbage, broccoli, cauliflower and eggs, and alcohol on the day before the test. They will also be asked to avoid candies and chewing gums on the morning of the test. The presence of halitosis will be recorded for VSC measurements ≥ 75 ppb.

Participants will be asked not to perform oral hygiene procedures from the evening before the examination.

S2. CONSORT 2010 checklist of information to include when reporting a randomized trial.

Section/Topic	Item No	Checklist item	Reported on page No
**Title and abstract**			
	1a	Identification as a randomised trial in the title	1
	1b	Structured summary of trial design, methods, results, and conclusions (for specific guidance see CONSORT for abstracts)	1
**Introduction**			
Background and objectives	2a	Scientific background and explanation of rationale	2
	2b	Specific objectives or hypotheses	2-3
**Methods**			
Trial design	3a	Description of trial design (such as parallel, factorial) including allocation ratio	3
	3b	Important changes to methods after trial commencement (such as eligibility criteria), with reasons	n.a.
Participants	4a	Eligibility criteria for participants	3
	4b	Settings and locations where the data were collected	3
Interventions	5	The interventions for each group with sufficient details to allow replication, including how and when they were actually administered	3
Outcomes	6a	Completely defined pre-specified primary and secondary outcome measures, including how and when they were assessed	4-5
	6b	Any changes to trial outcomes after the trial commenced, with reasons	n.a.
Sample size	7a	How sample size was determined	3
	7b	When applicable, explanation of any interim analyses and stopping guidelines	n.a.
Randomisation:			
Sequence generation	8a	Method used to generate the random allocation sequence	3
	8b	Type of randomisation; details of any restriction (such as blocking and block size)	3

Allocation concealment mechanism	9	Mechanism used to implement the random allocation sequence (such as sequentially numbered containers), describing any steps taken to conceal the sequence until interventions were assigned	3
Implementation	10	Who generated the random allocation sequence, who enrolled participants, and who assigned participants to interventions	3
Blinding	11a	If done, who was blinded after assignment to interventions (for example, participants, care providers, those assessing outcomes) and how	3
	11b	If relevant, description of the similarity of interventions	n.a.
Statistical methods	12a	Statistical methods used to compare groups for primary and secondary outcomes	5
	12b	Methods for additional analyses, such as subgroup analyses and adjusted analyses	5
**Results**			
Participant flow (a diagram is strongly recommended)	13a	For each group, the numbers of participants who were randomly assigned, received intended treatment, and were analyzed for the primary outcome	5-6
	13b	For each group, losses and exclusions after randomization, together with reasons	5-6
Recruitment	14a	Dates defining the periods of recruitment and follow-up	5
	14b	Why the trial ended or was stopped	5
Baseline data	15	A table showing baseline demographic and clinical characteristics for each group	n.a.
Numbers analysed	16	For each group, number of participants (denominator) included in each analysis and whether the analysis was by original assigned groups	n.a.
Outcomes and estimation	17a	For each primary and secondary outcome, results for each group, and the estimated effect size and its precision (such as 95% confidence interval)	6-9
	17b	For binary outcomes, presentation of both absolute and relative effect sizes is recommended	6-9
Ancillary analyses	18	Results of any other analyses performed, including subgroup analyses and adjusted analyses, distinguishing pre-specified from exploratory	6-9

Harms	19	All important harms or unintended effects in each group (for specific guidance see CONSORT for harms)	n.a.
**Discussion**			
Limitations	20	Trial limitations, addressing sources of potential bias, imprecision, and, if relevant, multiplicity of analyses	11-12
Generalisability	21	Generalisability (external validity, applicability) of the trial findings	9-11
Interpretation	22	Interpretation consistent with results, balancing benefits and harms, and considering other relevant evidence	9-11
**Other information**			
Registration	23	Registration number and name of trial registry	3
Protocol	24	Where the full trial protocol can be accessed, if available	3
Funding	25	Sources of funding and other support (such as supply of drugs), role of funders	12

S3. Inclusion and exclusion criteria

Inclusion criteria were:

- adult subjects aged 18 to 64 years,
- with at least 24 natural teeth (excluding third molars),
- gingival index and plaque index scores ≤2,
- and a stimulated salivary flow rate between 1.5 and 2.0 mL/min.

Exclusion criteria were:

- presence of systemic diseases,
- pregnancy or lactation,
- history of drug abuse,
- smoking habits,
- use of fixed orthodontic appliances,
- and allergies to any ingredients contained in the chewing gums used.

S4. Production and use of chewing gum.

All chewing gums used in the study were produced and supplied by Perfetti Van Melle. The sugar-free chewing gums (weight 2.1 g) were formulated with gum base (Gum Base Co., Via Nerviano, 25, 20045 Lainate, Italy), food-grade polyols, excluding xylitol (proprietary blend; manufactured by Roquette Frères S.A. and Cargill Srl, 1 Rue de la Haute Loge, 62136 Lestrem, France), food-grade intensive sweeteners (Ajinomoto Co., Inc, 1-15-1 Kyobashi, Chuo-ku, Tokyo 104-8315, Japan), flavors (Mondarom Selegroven AG, Via Maito 8, 6804 Bironico, Svizzera) and incorporated Heyndrickxia coagulans SNZ1969® (provided by Sanzyme Biologics Ltd, Sattva Signature Tower, H.No. 8-2-472/1/A/B/SF-3, Road No. 1, Banjara Hills, Hyderabad, 500034 Telangana, India). Initially, the gum base is melted at 50°C and combined with polyols, artificial intense sweeteners, and flavourings sequentially, achieving a homogeneous mixture. The freeze-dried probiotic biomass is incorporated as a final component below 50°C to preserve spore integrity and viability. After mixing, in the rolling and scoring system, a mass of gum is extruded into a thick slab, which is then worked into a thinner and thinner foil by a series of rollers. Finally, the foil is shaped into single pieces by one or more cutting rollers. These pieces undergo cooling in a conditioning room before being panned, and afterward, individual pieces are prepared for packaging. At the end of the production process, the amount of Colony Forming Units (CFUs) contained in the chewing gum was assessed according to the following protocol. The gum pieces in the bag were manually crushed and then processed in a Stomacher® (Stomacher® 3500 peristaltic homogenizer, Seward, West Sussex, United Kingdom) for 2 min. The resulting homogenized chewing gum mass was further serially diluted 1:10 in Maximum Recovery Diluent (MRD) buffer (GranuCult® prime Peptone salt solution - Maximum recovery diluent, Merck KGaA, Darmstadt, Germany). Aliquots of 100 µL were plated in GYEA-agar (glycerol 5 g/L, yeast extract 2 g/L, K2HPO4 1 g/L, bromocresol green 10 mL/L from a stock of 5 g/L, agar 15 g/L pH 5.5, Merck KGaA, Darmstadt, Germany). Part of the samples underwent via-ble count after pasteurization (incubation in a water bath at 90°C for 10 min) to quantify bacterial spores. The plates were incubated at 55°C for 72 h in anaerobic conditions, established by incubating the plates in AnaeroJar Oxoid 2.5 L jars (Thermo Fisher Scientific™, Waltham, Massachusetts, USA) containing Anaerocult™ A (Merck KGaA, Darmstadt, Ger-many). The procedures described were repeated for each sample in triplicate. CFUs were identified by morphology and colour and finally counted. The viable bacterial cell counts were expressed as colony-forming units /gram (CFU/g). At the end of the production process, the mean counts of probiotics in one pellet of chewing gum were 5 × 108 CFU. The control chewing gum was matched to the test gum in terms of shape, colour, and composition, and was produced using the same process, but it did not contain any probiotics

The study lasted a total of seven weeks. All enrolled subjects received instructions for at-home oral hygiene procedures, along with a manual toothbrush and a fluoride toothpaste (1450 ppm F) to be used throughout the study period. Participants were instructed not to use any mouthwash or other oral hygiene products aside from those provided by researchers. Additionally, participants were asked to avoid the use of antibacterial or antibiotic medications (topical or systemic), probiotics, chewing gum, and other xylitol-containing products during the study period. Participants requiring any of these products were instructed to notify the investigators and were subsequently withdrawn from the study. The experimental period was structured as shown in Figure 1: an initial two-week washout phase, followed by a four-week intervention period, and concluding with a one-week post-intervention stage. During the four-week intervention period, participants were instructed to consume the assigned chewing gum five times per day (after breakfast, mid-morning, after lunch, mid-afternoon, and in the evening after dinner), at least 30 minutes after brushing their teeth. To promote compliance and ensure correct product intake, participants were provided with blister packs containing the exact number of chewing gums required between two consecutive follow-up visits (n = 70).

Participants were instructed to return the empty blister packs at the subsequent visit to verify adherence to the intervention. In addition, they received a paper diary in which they were asked to record each chewing gum intake. Adherence to the study protocol, at-home oral hygiene practices, and the occurrence of any adverse effects were monitored at each evaluation using a specifically designed questionnaire.

S5. Dental plaque sampling and microbiome analysis

Dental plaque was collected from buccal and lingual tooth surfaces using sterile FLOQSwabs® (Copan Italia S.p.A., Brescia, Italy) and immediately transferred into tubes containing 1 mL of nucleic acid preservation medium (eNAT®, Copan Italia S.p.A., Brescia, Italy). Samples were stored at 4°C, transported within 2 h, and subsequently frozen at −80°C until processing.

DNA extraction was performed using the QIAsymphony DSP Virus/Pathogen Midi Kit® (Qiagen, Milan, Italy) according to the manufacturer's instructions. Microbial profiling was conducted by 16S rRNA gene sequencing targeting the V3–V4 region (primers 515F/806R) on an AVITI platform (Element Biosciences) using paired-end 2 × 300 bp chemistry at the Center for Omics Sciences (COSR, San Raffaele Hospital, Milan, Italy). Raw FASTQ files and run quality reports (FastQC) were provided by the sequencing facility. Sequence processing was performed in QIIME 2 (v2024.5). After demultiplexing, reads were quality filtered, denoised, merged, and chimera-checked using DADA2, yielding an ASV feature table. Quality filtering parameters were set based on per-base quality profiles. Alpha diversity was assessed using observed ASVs, Shannon index, Faith's phylogenetic diversity, and Pielou's evenness. Beta diversity was evaluated using Bray–Curtis, Jaccard, weighted UniFrac, and unweighted UniFrac metrics. Taxonomy was assigned using a Naive Bayes classifier trained on the SILVA 138/138.1 database (99% OTUs), trimmed to the V3–V4 region. Feature tables were aggregated to higher taxonomic levels (phylum to genus) for downstream analyses.

S6. Alpha-diversity analyses (previously reported in the primary publication arising from the same randomized clinical trial).

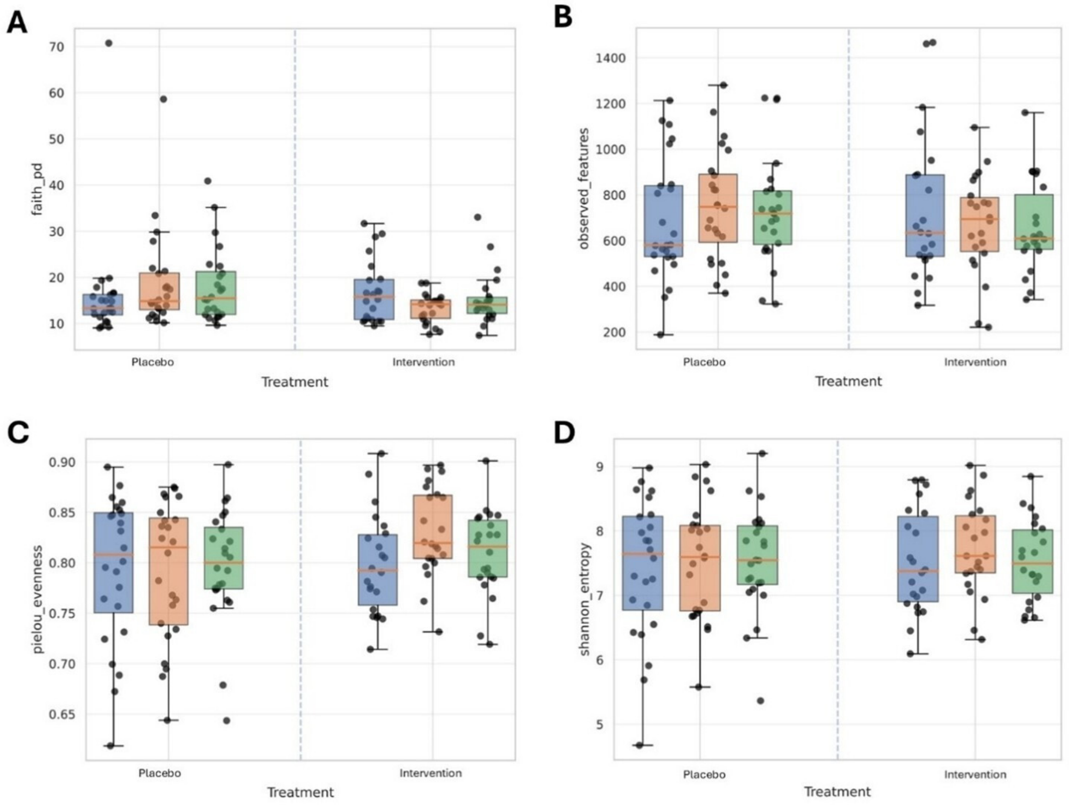

S7. Beta-diversity analysis (PCoA) (previously reported in the primary publication arising from the same randomized clinical trial).

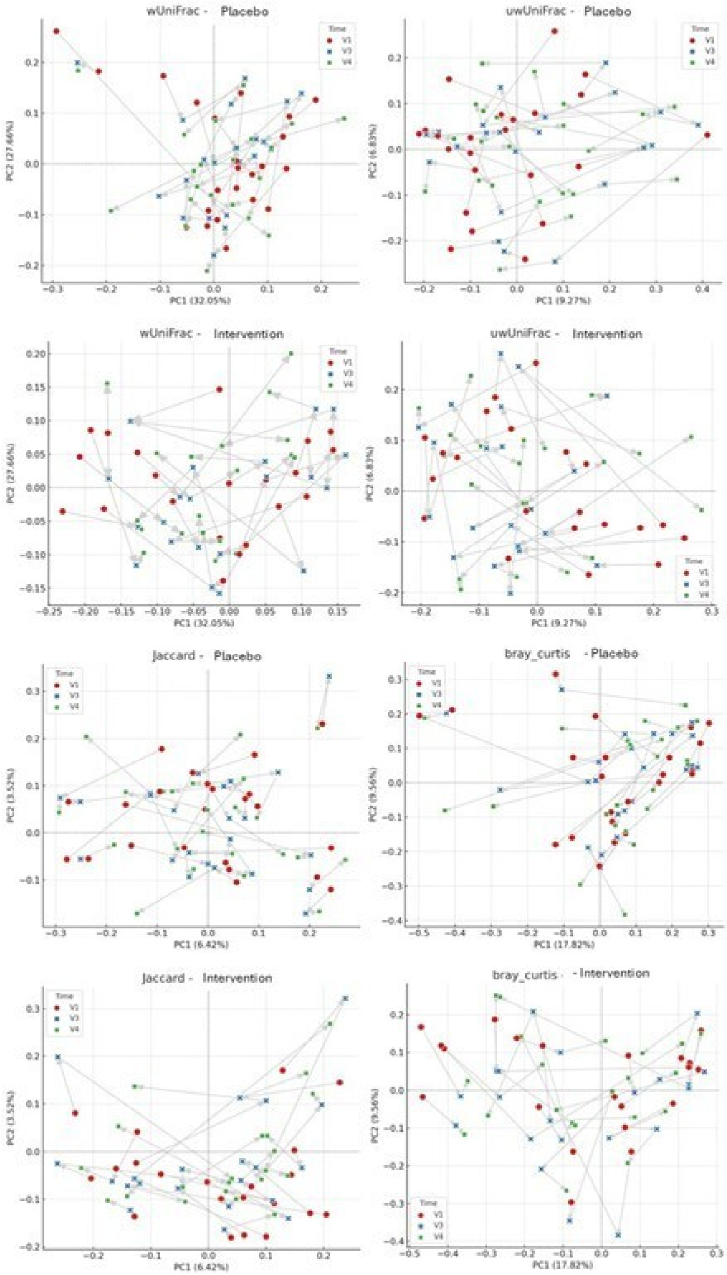

S8. Nominally VSCs-associated taxa.

Endpoint	Level	Nominally VSCs-associated taxa	Taxa nominally modified by the probiotic	Taxa consistent in direction
CH_3SH	Family	36	11	10
CH_3SH	Genus	64	9	7
CH_3SH	Species	38	4	4
H_2S	Family	74	27	25
H_2S	Genus	116	25	24
H_2S	Species	84	12	11

S9. Exploratory H_2S-associated taxa showing nominal treatment-related changes with coherent directionality. Taxa were selected among nominally significant features that were taxonomically interpretable, compatible with the oral ecosystem, and directionally consistent with their baseline association with H2S levels.

**Taxon**	**Δ time point**	**Δ median in PROBIOTIC group**	**Δ median in PLACEBO group**	**p-Value (**Mann-Whitney U test **)**	**Interpretation**
Leptotrichiaceae	T_2-T_0	−0.32	+0.49	0.015	↓ in probiotic group; ↑ in placebo group
Leptotrichiaceae	T_3-T_0	−0.39	+0.63	0.017	Effect maintained even at T_3
Leptotrichia	T_2-T_0	−0.29	+0.40	0.010	↓ in probiotic group; ↑ in placebo group
Leptotrichia	T_3-T_0	−0.35	+0.65	0.011	Effect maintained even at T_3
Kingella	T_2-T_0	−0.51	+0.93	0.021	↓ in probiotic group; ↑ in placebo group
Kingella	T_3-T_0	−0.38	+0.32	0.035	↓ in probiotic group; ↑ in placebo group
Actinomyces timonensis	T_2-T_0	+0.12	−0.08	0.021	↑ in probiotic group; ↓ in placebo group
Eubacterium sulci	T_2-T_0	+0.18	−0.09	0.010	↑ in probiotic group; ↓ in placebo group
Eubacterium sulci	T_3-T_0	+0.08	−0.16	0.010	↑ in probiotic group; ↓ in placebo group
Leptotrichia hofstadii	T_2-T_0	−0.53	+0.77	0.046	↓ in probiotic group; ↑ in placebo group

↑: increasing; ↓: reduction.
